# The impact of depression and antidepressant usage on primary biliary cholangitis clinical outcomes

**DOI:** 10.1371/journal.pone.0194839

**Published:** 2018-04-04

**Authors:** Abdel-Aziz Shaheen, Gilaad G. Kaplan, Wagdi Almishri, Isabelle Vallerand, Alexandra D. Frolkis, Scott Patten, Mark G. Swain

**Affiliations:** 1 Division of Gastroenterology and Hepatology, Department of Medicine, University of Calgary, Calgary, Alberta, Canada; 2 Snyder Institute for Chronic Diseases, Cumming School of Medicine, University of Calgary, Calgary, Alberta, Canada; 3 Departments of Community Health Sciences and Psychiatry, University of Calgary, Calgary, Alberta, Canada; Laval University, CANADA

## Abstract

**Background:**

Depression is prevalent in primary biliary cholangitis (PBC) patients. Our aims were to examine the effects of depression and antidepressants on hepatic outcomes of PBC patients.

**Methods:**

We used the UK Health Improvement Network database to identify PBC patients between 1974 and 2007. Our primary outcome was one of three clinical events: decompensated cirrhosis, liver transplantation and death. We assessed depression and each class of antidepressant medication in adjusted multivariate Cox proportional hazards models to identify independent predictors of outcomes. In a sensitivity analysis, the study population was restricted to PBC patients using ursodeoxycholic acid (UDCA).

**Results:**

We identified 1,177 PBC patients during our study period. In our cohort, 86 patients (7.3%) had a depression diagnosis prior to PBC diagnosis, while 79 patients (6.7%) had a depression diagnosis after PBC diagnosis. Ten-year incidence of mortality, decompensated cirrhosis, and liver transplantation were 13.4%, 6.6%, and 2.0%, respectively. In our adjusted models, depression status was not a predictor of poor outcomes. After studying all classes of antidepressants, using the atypical antidepressant mirtazapine after PBC diagnosis was significantly protective (Adjusted HR 0.23: 95% CI 0.07–0.72) against poor liver outcomes (decompensation, liver transplant, mortality), which remained statistically significant in patients using UCDA (HR 0.21: 95% CI 0.05–0.83).

**Conclusions:**

In our study, depression was not associated with poor clinical outcomes. However, using the antidepressant mirtazapine was associated with decreased mortality, decompensated cirrhosis and liver transplantation in PBC patients. These findings support further assessment of mirtazapine as a potential treatment for PBC patients.

## Introduction

Primary biliary cholangitis (PBC) is an autoimmune disease characterized by immune-mediated destruction of small hepatic bile ducts[1]. The bile acid ursodeoxycholic acid (UDCA) has been the only available therapy for PBC for over two decades. Unfortunately, 30% to 40% of patients with PBC do not fully respond to UDCA and are at risk of progressing to cirrhosis, liver failure, and death[2].

PBC is commonly associated with significant systemic symptoms[1, 3]. The prevalence of depressive symptoms, such as depressed mood, impaired cognition, social withdrawal and loss of interest among PBC patients, is very high (30–45%)[4–7]. Interestingly, significantly fewer of these patients are typically assigned a formal depression diagnosis based on Diagnostic and Statistical Manual IV (DSM-IV) criteria[4]. In the context of clinical trials, depression is often measured with standardized self-reported questionnaires. However, significant overlap can exist in many of these questionnaires between disease-related symptoms (eg. fatigue, loss of interest) and neurovegatative symptoms of depression[4–6]. This overlap in turn can lead to the reporting of a higher frequency of depressive symptoms amongst patient groups compared to a lower frequency of patients with formal diagnoses of depression. Similar observations have been made in PBC[4]. Importantly, antidepressant usage is common among PBC patients due to the high prevalence of both depression and depressive symptoms [8].Over the last decade, depression has been recognised as a risk factor for poor outcomes in various autoimmune and chronic inflammatory conditions[9–14]. Remarkably, depression was also identified as a risk factor for developing autoimmune diseases in a large population based study[15]. Moreover, depression was associated with increased mortality and poor clinical outcomes in decompensated cirrhosis patients [16, 17].

Antidepressants can impact immunity and modulate inflammatory responses[18–20]. In addition, recent animal model studies have shown that serotonin can regulate hepatic immunity and decrease tissue damage [21–23]. While antidepressant usage in liver transplant patients decreased rates of cellular rejection, the clinical impact of antidepressant usage on a variety of liver conditions has not been established [24].

The effect of depression, or antidepressants, on disease outcomes in PBC patients is unknown. Therefore, we used data extracted from a large clinical database to examine the effect of depression and antidepressant medication treatment on hepatic outcomes of PBC patients, including hepatic decompensation, transplantation, and death.

## Methods

### Study design and patient data source

We conducted a cohort study using data from The Health Improvement Network (THIN). THIN is one of the largest medical databases in the UK[25], consisting of prospectively gathered electronic medical records from over 11.1 million patients[25]. Patients registered in THIN have demographic and mortality distributions comparable to the general UK population[26, 27]. Data from participant general practitioners across the UK is exported to the THIN administrators and database, which is updated every 3 months[28]. The THIN database records demographic data and clinical events using Read codes, prescription medications, and laboratory values[29]. The validity of the THIN database coding system (Read Codes) has been assessed in previous studies [30–33].

### Study population and outcomes

**Fig 1:**
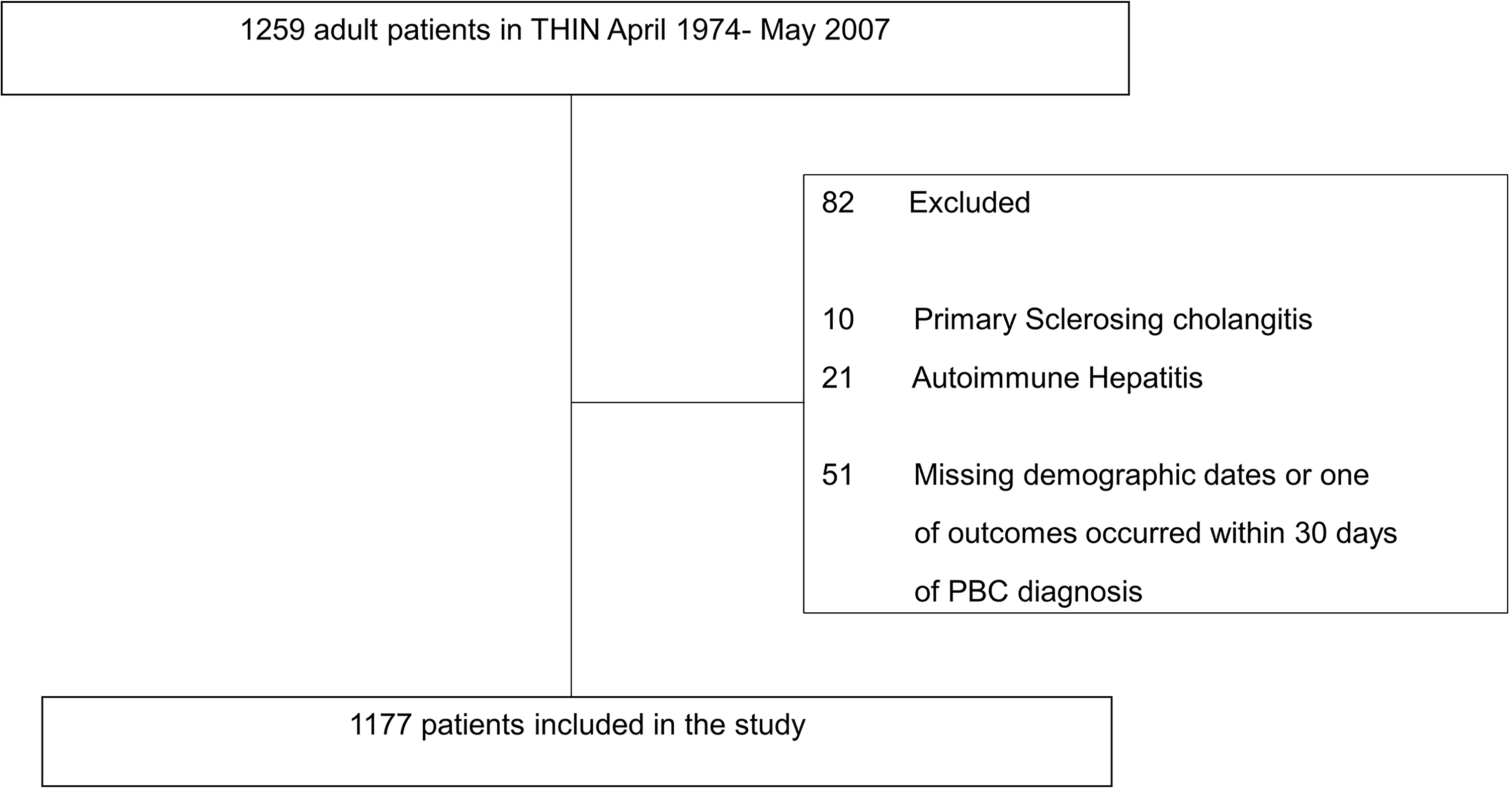
Flow diagram of identified PBC patients in The Health Improvement Network between April 1974 and May 2007.

We identified patients >18 years with PBC Read code “J6160” from April 1974 until May 2007. This cohort was followed until May 1, 2012. Patients with overlap syndromes with autoimmune hepatitis or primary sclerosing cholangitis were excluded. The first code of PBC identified in the THIN database was considered the index (i.e. diagnosis) date. Fig 1 provides a flow diagram illustrating the study population selection. The primary outcome was first occurrence of one of three events following the diagnosis of PBC: (1) decompensation of cirrhosis (ascites, spontaneous bacterial peritonitis, hepatic encephalopathy, hepatorenal syndrome, variceal bleeding, jaundice, or hepatocellular carcinoma) which were all defined by using Read codes; (2) liver transplantation; or (3) death. Patients who did not develop an outcome during the follow-up period were censored on May 1, 2012. All accessed data were fully anonymized.

### Exposure variables of interest

Read codes were used to identify patients diagnosed with depression (i.e., at least one code for a depressive disorder). Read codes associated with only symptoms of depression, bipolar disorder, mania or hypomania were excluded[34]. Depression was coded as follows: (1) never diagnosed with depression; (2) past depression diagnosis prior to 90 days from PBC diagnosis; and (3) current depression diagnosis if a diagnosis was made within 90 days before or after a PBC diagnosis. Using THIN database therapy files, we assessed the impact of antidepressant medications on PBC patient outcomes (mortality, decompensation, and liver transplant).

For each antidepressant medication use was defined as follows: (1) never used the medication; (2) past use defined as a previous code for medication use prior to 90 days of PBC diagnosis; and (3) current medication user if the medication code was identified within 90 days before or after a PBC diagnosis. We assessed the effect of each of the following typical and atypical antidepressant medications on study outcomes separately, as follows: Atypical antidepressants included agomelatine, mirtazapine and bupropion; Typical antidepressants included the following groups: (a) Selective Serotonin Reuptake Inhibitors (SSRI): citalopram, escitalopram, fluoxetine, fluvoxamine, paroxetine and sertraline; (b) Selective-Norepinephrine Reuptake Inhibitors (SNRI): desvenlafaxine, duloxetine, milnacipran and venlafaxine; (c) serotonin modulators: nefazodone, trazodone and vilazodone; (d) tricyclics and tetracyclics: amitriptyline, amoxapine, clomipramine, desipramine, doxepin, imipramine, maprotiline, nortriptyline and protriptyline, trimipramine; (e) monamine oxidase inhibitors: isocarboxazid, phenelzine, selegiline and tranylcypromine.

### Study covariates

We assessed age at PBC diagnosis, sex, and the presence of coexisting liver disease (defined as having chronic liver disease; either viral, alcoholic, or fatty liver disease; yes/no). Smoking status was classified (current, former, never smoked, or unknown status), and alcohol consumption was classified (current, former, never consumed alcohol, or unknown status), both at the time of PBC diagnosis. Ursodeoxycholic acid (UDCA) usage (ever/never recorded) was also determined.

### Data analysis

In our primary analysis we assessed demographic, clinical, and medication variables in the PBC cohort according to depression status. Where appropriate, we used the Fisher Exact test or Chi-Square test for categorical data, and the Kruskal-Wallis or Student’s t-test for continuous data. Univariate analysis using Log Rank test and multivariate Cox Proportional Hazards models were used to assess the impact of depression or antidepressant medications on PBC outcomes as defined by death, decompensated cirrhosis, or liver transplant. Each outcome was studied separately in sensitivity analyses. In all models, we adjusted for age, sex, UDCA usage, and depression status. We assessed for interaction between depression status and antidepressant usage, if both were included in the same model. Estimates were reported as hazard ratios (HR) and accompanying 95% confidence intervals (CIs). The proportional hazard model assumption was tested and not violated in any models.

### Sensitivity analysis

To assess the validity of our findings, we restricted the study population to PBC patients only using UDCA. Different survival analyses were performed to identify survival predictors in each of our study outcomes separately (cirrhosis decompensation, liver transplant, mortality). All analyses were performed using Stata 14.1 (StataCorp, College Station, TX) using alpha of 0.05. Both the Conjoint Health Research Ethics Board at the University of Calgary and The Scientific Review Committee of THIN approved the study protocol (ID:16THIN031).

## Results

### Cohort characteristics

1,177 PBC patients were identified from April 1974 to May 2007. In this cohort, 86 patients (7.3%) were diagnosed with major depressive disorder (MDD) prior to the diagnosis of PBC, while 79 patients (6.7%) had a depression diagnosis after their PBC diagnosis. Demographic and clinical characteristics of PBC patients according to depression status are shown in Table 1. PBC patients with prior or current depression were younger than those without depression (median age 59 and 58 versus 63 years, P = 0.009), and were more commonly female (93% and 96% versus 87%, P = 0.02). Prevalence of coexisting liver disease was similar among the three groups (P = 0.19), Table 1.

**Table 1:** Patients characteristics according to depression status.

Characteristic	PBC- No depression cohort n = 1,012 (86.0%)	PBC- Previous depression cohort n = 86 (7.3%)	PBC- Current depression cohort n = 79 (6.7%)	*P*-value
Age at diagnosis	63 (53–72)	59 (50–71)	58 (47–69)	0.009
Female Gender	87.1% (881)	93.0% (80)	96.2% (76)	0.02
Smoking				
Current	16.9% (171)	19.8% (17)	20.3% (16)	
Ex-smoker	36.0% (364)	45.4% (39)	32.9% (26)	0.32
No smoking	41.1% (416)	32.6% (28)	43.0% (34)	
Unknown	6.0% (61)	2.3% (2)	3.8% (3)	
Alcohol				
Current	50.1% (507)	54.7% (47)	48.1% (38)	
Ex-usage	25.6% (259)	29.1% (25)	29.1% (23)	0.48
Never	10.0% (101)	9.3% (8)	6.3% (5)	
Unknown	14.3% (145)	7.0% (6)	16.5% (13)	
Coexisting Liver disease	4.6% (46)	8.1% (7)	7.6% (6)	0.19
URSO usage	67.9% (687)	60.5% (52)	68.4% (54)	0.36
Ascites	3.7% (37)	1.2% (1)	1.3% (1)	0.27
SBP	0.3% (3)	0	0	0.78
Hepatic encephalopathy	0.6% (7)	0	0	0.61
Esophageal Varices	8.8% (89)	4.7% (4)	7.6% (6)	0.40
HRS	0.2% (2)	0	0	0.85
HCC	1.0% (10)	0	0	0.44
Jaundice	0.4% (4)	0	1.3% (1)	0.43
Follow up period, in months	91 (56–141)	75 (47–113)	133 (85–172)	<0.001
Decompensated Cirrhosis	12.6% (127)	5.8% (5)	8.9% (7)	0.13
Liver transplant	3.9% (39)	1.2% (1)	2.5% (2)	0.24
Death	27.1% (274)	19.8% (17)	26.6% (21)	0.34
Antidepressants				
Current usage	24.6% (249)	26.7% (23)	82.3% (65)	<0.001
Previous usage	11.1% (112)	45.4% (39)	7.6% (6)	
Antidepressants subgroups:				
SSRI				
Current usage	11.8% (119)	14.0% (12)	65.8% (52)	<0.001
Previous usage	7.5% (76)	50.0% (43)	17.7% (14)	
SNRI				
Current usage	1.6% (16)	2.3% (2)	12.7% (10)	<0.001
Previous usage	0.7% (7)	4.7% (4)	1.3% (1)	
Atypical				
Current usage	2.8% (28)	4.7% (4)	7.6% (6)	<0.001
Previous usage	0.9% (9)	9.3% (8)	1.3% (1)	
Serotonin Modulators				
Current usage	1.0% (10)	2.3% (2)	2.5% (2)	<0.001
Previous usage	0.8% (8)	2.3% (2)	1.3% (1)	
Tricyclic/ Tetracyclic				
Current usage	14.5% (147)	10.5% (9)	21.5% (17)	<0.001
Previous usage	8.8% (89)	33.7% (29)	13.9% (11)	
MOI				
Current usage	0	0	0	
Previous usage	0	0	0	
Mirtazapine				
Current usage	2.7% (27)	3.5% (3)	6.3% (5)	<0.001
Previous usage	0.4% (4)	8.1% (7)	0	

Data is presented as percentage and numbers for categorical data or median and interquartile range for continuous data.

URSO, ursodeoxycholic acid; SBP, spontaneous bacterial peritonitis; HRS, hepatorenal syndrome; HCC, hepatocellular carcinoma; SSRI, selective serotonin reuptake inhibitors; SNRI, serotonin and norepinephrine reuptake inhibitors; MOI, monoamine oxidase inhibitors.

### Clinical outcomes

Approximately 70% of PBC patients were prescribed UDCA, which did not differ by depression status (see Table 1). The cumulative incidence of mortality at 3, 5, and 10 years was 6.5%, 13.4%, and 25.4%, respectively. The cumulative incidence of decompensated cirrhosis and liver transplant at 3, 5, and 10 years were 4.1%, 6.6%, 13.0% and 0.1%, 2.0%, 4.0%, respectively. During a median follow-up of 92 months (range: 58–143 months), overall decompensated cirrhosis, liver transplant, and mortality rates were similar amongst our study groups (overall mortality: 27.1% for non-depression cohort, 19.8% for previously diagnosed with depression, and 26.6% for currently depressed patients, P = 0.34).

### Antidepressant medication usage

Usage of anti-depressant medications was variable amongst our study groups (see Table 1). Interestingly, among PBC patients without a diagnosis of depression, 24.6% were using antidepressants after their PBC diagnosis, while 11.1% used antidepressants prior to their PBC diagnosis. Mirtazapine was prescribed at a lower rate for patients with no history of depression (2.7%) or with a previous depression diagnosis (3.5%), compared to those with a current depression diagnosis (6.3%), P<0.001. Details on anti-depressant usage are shown in Table 1.

### Impact of depression and antidepressants on PBC patient survival

**Fig 2:**
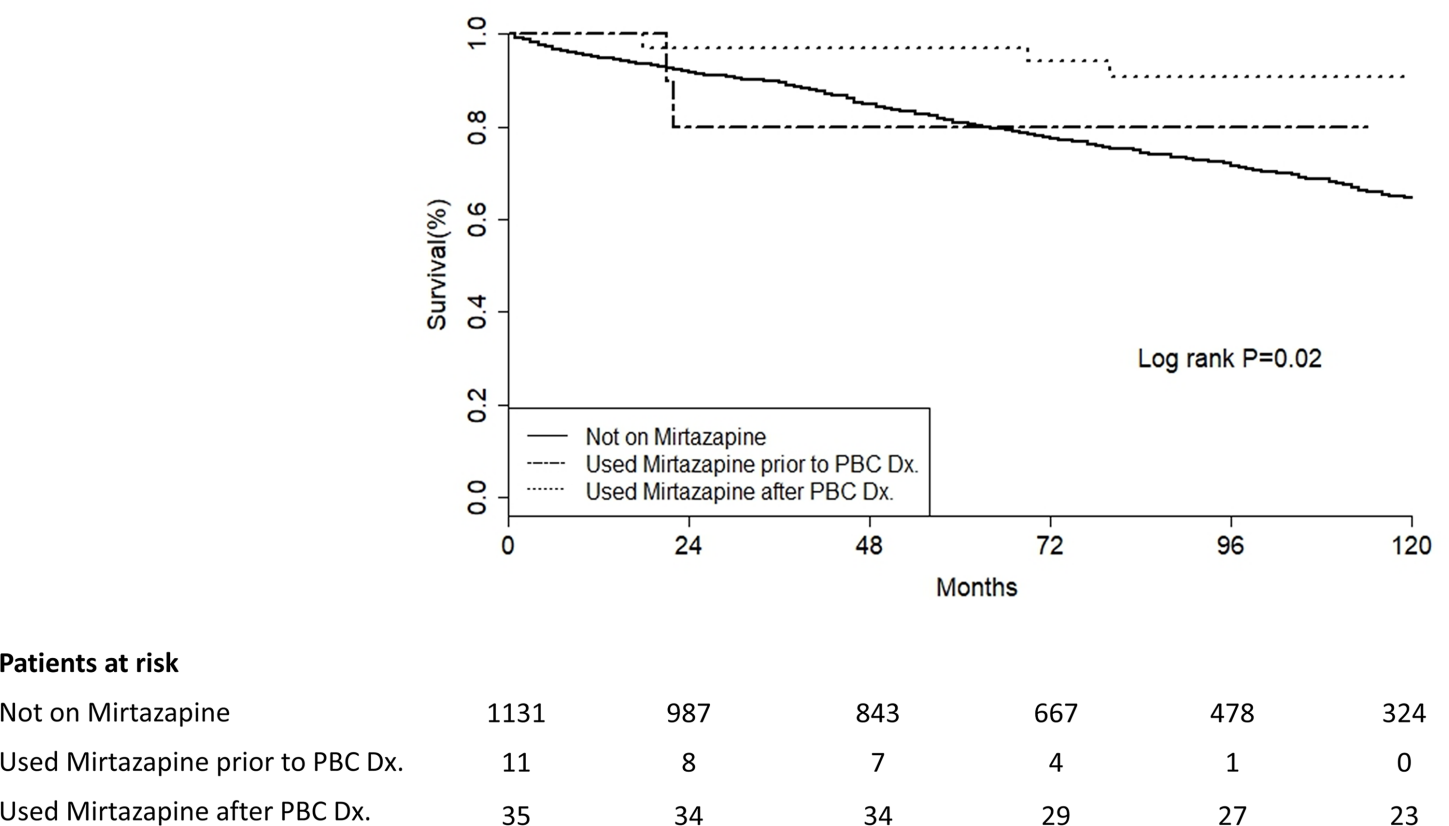
Kaplan-meier curves indicating 10-year decompensation, liver transplant and mortality free survival among PBC patients according to mirtazapine usage.

PBC patients with depression were less likely to die, compared to PBC patients without depression (HR 0.52: 95% CI 0.31–0.87). To examine this association further and to assess the role of antidepressants on this protective effect, we used adjusted models for age, gender, UDCA usage, alcohol intake, depression status, and assessing for each of the aforementioned anti-depressant classes and drugs separately. Mirtazapine was the only antidepressant independently associated with a decreased risk of decompensated cirrhosis, transplantation, or death (Table 2). In our adjusted models, using mirtazapine after PBC diagnosis was significantly protective (Adjusted HR 0.23: 95% CI 0.07–0.72) against poor outcomes (decompensation, liver transplant, mortality). Interestingly, current depression status was not a significant predictor of poor outcomes (Adjusted HR 0.65: 0.38–1.12). There was no interaction between depression status and mirtazapine usage in our models. See Table 2 and Fig 2.

**Table 2:** Predictors of 10-year decompensation, liver transplant and mortality free survival in PBC cohort.

Variable	Univariate HR	Multivariate model-1 (include age, gender, alcohol, URSO, depression, antidepressants)	Multivariate model-2^$^ (include age, gender, alcohol, URSO, depression, mirtazapine)
Age at diagnosis	1.05 (1.04–1.06)	1.05 (1.04–1.06)	1.05 (1.04–1.06)
Female Gender	0.83 (0.60–1.14)	0.93 (0.67–1.29)	0.92 (0.67–1.28)
Smoking			
Current vs other categories	1.03 (0.77–1.38)		
Alcohol			
Current vs other categories	0.77 (0.61–0.96)	0.82 (0.66–1.03)	0.82 (0.66–1.03)
Coexisting Liver disease	1.30 (0.82–2.08)		
Depression diagnosis			
None	Ref	Ref	Ref
Prior to PBC diagnosis	0.82 (0.51–1.30)	0.81 (0.50–1.31)	0.84 (0.52–1.35)
Current	0.52 (0.31–0.87)	0.65 (0.38–1.12)	0.60 (0.35–1.00)
URSO usage	0.80 (0.64–1.00)	1.00 (0.79–1.26)	1.00 (0.79–1.27)
Antidepressants			
None	Ref	Ref	
Prior to PBC diagnosis	1.04 (0.74–1.46)	1.10 (0.78–1.57)	
Current	0.68 (0.52–0.87)	0.79 (0.60–1.04)	
SSRI			
None	Ref		
Prior to PBC diagnosis	0.73 (0.49–1.09)		
Current	0.76 (0.56–1.03)		
SNRI			
None	Ref		
Prior to PBC diagnosis	1.55 (0.58–4.17)		
Current	0.74 (0.35–1.57)		
Atypical			
None	Ref		
Prior to PBC diagnosis	0.71 (0.23–2.23)		
Current	0.27 (0.10–0.73)		
Serotonin Modulators			
None	Ref		
Prior to PBC diagnosis	0.31 (0.04–2.17)		
Current	0.46 (0.11–1.85)		
Tricyclic/ Tetracyclic			
None	Ref		
Prior to PBC diagnosis	1.20 (0.85–1.69)		
Current	0.74 (0.53–1.02)		
Mirtazapine*			
None	Ref		Ref
Prior to PBC diagnosis	0.84 (0.21–3.39)		0.94 (0.23–3.94)
Current	0.22 (0.07–0.69)		0.23 (0.07–0.72)
Fluoxetine*			
None	Ref		
Prior to PBC diagnosis	0.83 (0.50–1.37)		
Current	0.58 (0.36–0.92)		

* After adjusting for age at diagnosis, female gender, using of URSO, depression status, and alcohol intake. Fluoxetine was not significant while Mirtazapine remained significant in multivariate model

^$^ There was no interaction between Mirtazapine and depression status.

URSO, ursodeoxycholic acid; SSRI, selective serotonin reuptake inhibitors; SNRI, serotonin and norepinephrine reuptake inhibitors.

### Sensitivity analysis

First, we limited our cohort to only patients who were using UDCA (n = 793, 67.4%). In adjusted Cox Regression models, current usage of mirtazapine was protective against decompensated cirrhosis, liver transplant, and mortality (Adjusted HR 0.21: 95% CI 0.05–0.83). We assessed mirtazapine as an independent predictor for each of our outcomes separately (cirrhosis decompensation, liver transplant, and mortality). Current usage of mirtazapine was a significant predictor of decreased mortality (adjusted HR 0.22: 95% CI 0.05–0.89), but not significantly protective for liver decompensation (adjusted HR 0.22: 95% CI 0.03–1.60). None of the PBC patients who had ever used mirtazapine required liver transplant in our cohort.

## Discussion

This is the first study to describe the effect of depression and antidepressant usage on clinical outcomes of PBC patients. The current mainstay of medical treatment for PBC is UDCA[1]. However, 30–40% of PBC patients fail to respond to UDCA treatment[1, 2] and are at risk for disease progression to cirrhosis, liver failure, transplantation or death[2]. We used a large clinical database to study the effect of depression and antidepressants usage in a cohort of 1,177 PBC patients.

In our cohort of PBC patients, 14% had been diagnosed with depression (with ~7% having a depression diagnosis after their PBC diagnosis). The depression rate in our PBC cohort is consistent with previously reported rates of depression amongst PBC patients. In Al-harthy *et al*, the prevalence of depression among PBC patients was 12%[8], while Os *et al* reported a lower rate of 4% (95%CI 0–15%). Although the latter study included a small number of patients (PBC patients n = 55), it was the only study to validate the prevalence of depression among PBC patients with depressive symptoms (38%) according to DSM-IV criteria[4]. Depressive symptoms are common amongst PBC patients, likely due to the overlap of depression and disease-related symptoms such as fatigue within this population[3, 5]. In fact, fatigue has been reported in up to 80% of PBC patients [5–7]. Although depression is more prevalent in PBC than in the general population[35], no detailed study evaluated the impact of depression on PBC natural history. As for antidepressant usage, only one study assessed the effect of the antidepressant fluvoxamine on fatigue in PBC patients[36]. Nonetheless, no study has evaluated the effect of antidepressants on PBC clinical outcomes.

Amongst our PBC cohort, patients who suffered from previous or current depression were younger than patients with no history of depression (median age 59, 58 vs. 63). This finding is consistent with previous studies[4, 5]. Although the difference between groups was not clinically significant, one possible explanation is that younger patients are more likely to seek medical care and are subjected to a diagnosis bias. Moreover, PBC patients complaining of fatigue appear to be younger than older PBC patients[8]. Therefore, younger PBC patients are more likely to be exposed to further medical evaluation and subsequently diagnosed with depression.

In our patient cohort, the risk of death 10 years following PBC diagnosis was 25%. This high risk of death is consistent with previously published cohorts of PBC patients[1, 2, 37] and was associated with the occurrence of decompensated cirrhosis and transplantation. We explored the potential therapeutic effect of antidepressants on PBC prognosis following our observation that PBC patients with depression were less likely to die, compared to PBC patients without depression. Interestingly, after adjusting for antidepressant usage, the effect of depression on adverse liver outcomes was no longer observed. However, we found that use of the antidepressant mirtazapine had a significant protective effect against poor outcomes in our PBC patient cohort. Specifically, mirtazapine was responsible for a striking greater than four-fold protective effect against liver decompensation, transplantation, or death, which was robust following adjustment for potential confounders including age, sex, and alcohol use. The effect of mirtazapine was independent of UDCA use, as the association was consistent following adjustment for UDCA use in the Cox proportional hazard model. Moreover, our sensitivity analysis, that restricted the study population to patients with PBC taking UDCA, showed that mirtazapine’s protective effect was consistent in this cohort. Such a protective effect was not observed with any other antidepressant. While Fluoxetine had a protective effect in univariate analysis, this effect was not observed in our adjusted models.

Although mirtazapine was originally approved for the clinical treatment of depression, its’ unique pharmacological profile has facilitated widespread clinical use to treat numerous other clinical disorders, including sleep disturbance, addiction, and anxiety[38, 39]. Consistent with this, the majority of PBC patients in our cohort who were prescribed mirtazapine did not have a coexisting diagnosis of depression.

Mirtazapine exhibits a complex pharmacology, having both central and peripheral effects[38]. It can act as a 5HT2_A_ and 5HT2_B_ serotonin receptor antagonist, a 5HT2_C_ serotonin receptor inverse agonist, and an antagonist for 5HT3 and histamine (H1) receptors[38]. Therefore, given its’ complex pharmacology, mirtazapine could plausibly improve hepatic outcomes in PBC patients through a number of potential mechanisms involving serotonin receptor modulation or inhibition of histamine effects. In a number of animal models serotonin has been shown to regulate hepatic immunity, altering inflammatory responses and associated tissue damage that ultimately leads to progressive liver fibrosis[21–23]. Serotonin has been linked to the regulation of hepatocyte proliferation during liver regeneration via activation of 5HT_2_ receptors[40], and to enhanced tissue repair in hepatic ischemia/reperfusion injury[41]. It also has been involved in inhibiting cholangiocyte proliferation and cholestasis via 5HT1_A_ receptor activation[42], and shifting tissue macrophages towards a more anti-inflammatory phenotype[43]. In contrast, serotonin worsens experimental viral hepatitis[44] and contributes to disease progression in non-alcoholic fatty liver disease[41]. Importantly, platelets represent a rich source of serotonin and platelets significantly accumulate in the liver of PBC patients[45]. Therefore, mirtazapine modulation of serotonergic signaling in the liver of PBC patients could significantly alter hepatic immunity, which in turn could improve clinical outcomes, as observed in our patient cohort.

The protective effect of mirtazapine in PBC patients was supported by the large patient sample size and magnitude of the risk estimate. However, several limitations should be considered. We used a clinical database based on general practitioners’ electronic medical records, which raises the potential for misclassification error of codes used to define the study population and outcomes. Misclassification errors were evaluated with sensitivity analyses that mandated multiple codes to define PBC[46], and a sensitivity analysis that restricted the population to those with a code for PBC and a prescription for UDCA. In our study, we could not evaluate the biochemical response to UDCA as this data is not included in THIN. To minimize the effect of this limitation, we performed a sensitivity analysis limiting our cohort to patients who were treated with UDCA. We feel that our findings support future prospective studies evaluating the impact of mirtazapine in PBC patients according to UDCA biochemical response. In addition, an epidemiological association cannot prove causality or explain the biological mechanism of mirtazapine’s effect in PBC. Therefore, future studies should explore potential mechanisms underlying this beneficial impact of mirtazapine in PBC patients.

In conclusion, we have described the effect of depression and antidepressant usage in a large PBC patient cohort. We identified that use of the atypical antidepressant mirtazapine was associated with decreased mortality, reduced need for liver transplantation, and lower rates of decompensated cirrhosis amongst our PBC patients. This protective association of mirtazapine was independent of the role of depression, prescription of other antidepressants, or the use of UDCA. We suggest that these findings support the examination of mirtazapine as a potential novel therapy for PBC patients.
